# Characterisation of human milk bacterial DNA profiles in a small cohort of Australian women in relation to infant and maternal factors

**DOI:** 10.1371/journal.pone.0280960

**Published:** 2023-01-25

**Authors:** Azhar S. Sindi, Ali S. Cheema, Michelle L. Trevenen, Donna T. Geddes, Matthew S. Payne, Lisa F. Stinson

**Affiliations:** 1 Division of Obstetrics and Gynaecology, School of Medicine, The University of Western Australia, Perth, Western Australia, Australia; 2 College of Applied Medical Sciences, Umm Al-Qura University, Makkah, Saudi Arabia; 3 School of Molecular Sciences, The University of Western Australia, Perth, Western Australia, Australia; 4 Centre for Applied Statistics, The University of Western Australia, Perth, Western Australia, Australia; 5 Women and Infants Research Foundation, Perth, Western Australia, Australia; University of Illinois Urbana-Champaign, UNITED STATES

## Abstract

Human milk is composed of complex microbial and non-microbial components that shape the infant gut microbiome. Although several maternal and infant factors have been associated with human milk microbiota, no study has investigated this in an Australian population. Therefore, we aimed to investigate associations between human milk bacterial composition of Australian women and maternal factors (body mass index (BMI), mode of delivery, breast pump use, allergy, parity) and infant factors (sex, mode of feeding, pacifier use, and introduction of solids). Full-length 16S rRNA gene sequencing was used to characterise milk bacterial DNA profiles. Milk from mothers with a normal BMI had a higher relative abundance of *Streptococcus australis* than that of underweight mothers, while milk from overweight mothers had a higher relative abundance of *Streptococcus salivarius* compared with underweight and obese mothers. Mothers who delivered vaginally had a higher relative abundance of *Streptococcus mitis* in their milk compared to those who delivered via emergency caesarean section. Milk of mothers who used a breast pump had a higher relative abundance of *Staphylococcus epidermidis* and *Streptococcus parasanguinis*. Milk of mothers whose infants used a pacifier had a higher relative abundance of *S*. *australis* and *Streptococcus gwangjuense*. Maternal BMI, mode of delivery, breast pump use, and infant pacifier use are associated with the bacterial composition of human milk in an Australian cohort. The data from this pilot study suggests that both mother and infant can contribute to the human milk microbiome.

## Introduction

Human milk (HM) is made up of nutritive components, immune factors, and microbial communities [1–3]. It contributes to seeding of the infant gut [4–8] and oral cavity [9] microbiomes. *Bifidobacterium breve*, *B*. *adolescentis*, *B*. *dentium*, *B*. *infantis*, *B*. *longum*, *B*. *bifidum*, *B*. *angulatum*, *Staphylococcus epidermidis*, and *Veillonella parvula* have been reported among the shared bacterial species between HM and the infant gut microbiome [4, 6, 10, 11], while *S*. *epidermidis*, *S*. *auricularis*, *Streptococcus parasanguinis/gordonii*, *S*. *mitis/oralis*, and *S*. *salivarius* have been reported to be shared between HM and the infant oral microbiome [9]. Infant gut bacterial communities are important for immune development [12, 13] and may modify the risk of developing early-life [14, 15] as well as later-life diseases [16, 17]. Thus, HM microbial communities likely have important implications for infant health.

The composition of the HM microbiota varies between individuals [18]. Metataxonomic and metagenomic studies have revealed that *Staphylococcus* spp. and *Streptococcus* spp. are consistently present and highly abundant in HM [19–25], whereas the presence and abundance of other bacterial species are variable between individuals and populations [26]. Early-life maternal and infant factors have been associated with the bacterial composition of HM. Maternal factors such as mode of delivery [27–35], body mass index (BMI) [27, 30, 35–38], and breast pump use [34, 39] have been reported to be associated with HM bacterial profiles. Infant factors such as gestational age at delivery [28], sex [34, 38], and feeding method [34, 39–42] have also been reported to be associated with HM bacterial profiles.

While previous studies have sought to identify associations between maternal and infant factors and the HM microbiome in various populations, to date, no study has assessed these in an Australian setting. This is important, as the HM microbiome [26, 30, 32, 35, 43, 44], and the human microbiome [45–47] more generally, have been shown to vary between geographically distinct populations (due to a combination of genetic, local environment, and dietary factors). Being an isolated island nation, it is important to characterise the HM microbiota and describe any associated maternal and infant factors. This may aid in identifing potential avenues to alter HM microbial profiles in a manner that supports infant health.

In this pilot study, we analysed the milk microbiota of Western Australian women. We sought to determine the influence of maternal and infant factors on HM bacterial profiles in this population. Further, we have improved upon previous studies in this field by utilising full-length 16S rRNA gene sequencing, allowing deeper taxonomic resolution.

## Materials and methods

### Study population

Twenty nine predominantly breastfeeding women (those using HM as the main source of infant nourishment), with healthy infants aged 3–83 weeks and no nipple infection or nipple pain were recruited for this study. All mothers provided written informed consent prior to participation. Ethical approval was obtained from The University of Western Australia’s Human Research Ethics Committee (RA/4/1/2369).

### Maternal and infant demographic data collection

Data regarding maternal BMI, mode of delivery, maternal allergies, parity, infant sex, and pacifier use were collected via an online questionnaire. Mothers who self-reported having an allergic skin reaction or allergy to any food, medication, or animal were classified as having an allergy. Participants were assigned into one of the following BMI categories: underweight if less than 18.5, normal if between 18.5–24.9, overweight if between 25.0–29.9, Obesity class I if between 30.0–34.9, Obesity class II if between 35.0–39.9, and Obesity class III if above 40. Data regarding infant formula and solid intake, nipple pain, and breast pump use were collected at the time of sample collection.

### Sample collection

Milk samples were collected aseptically using a Symphony electric breast pump (Medela AG, Baar, Switzerland) with a sterile pump kit (microwave steam sterilised). To reduce contamination from the skin, participants cleaned the nipple and areola of the expressing breast with chlorhexidine wipes followed by rinsing with sterile saline and drying with sterile gauze. Up to 10 mL of post-milk ejection milk was collected directly into a sterile 50mL tube. Milk samples were kept on ice and immediately transported to the laboratory where they were aliquoted and stored at -80°C until DNA extraction.

### DNA extraction

Milk samples (1 mL) were centrifuged at 40,000 x g for 5 min at 4°C and the supernatant and fat were removed. DNA was extracted from the cell pellet using the MagAttract Microbial DNA Kit (QIAGEN) on the Kingfisher Flex platform according to the manufacturer’s instructions. Two negative extraction controls were processed alongside the samples. The negative extraction controls consisted of 1 ml of sterile DNA-free water (Integrated DNA Technologies, Queenstown, Singapore) were placed at the centre of the 96-well extraction plate.

### 16S rRNA gene amplification and PacBio HiFi sequencing

PCR amplification and PacBio High-Fidelity (HiFi) sequencing was performed as previously described [48]. Briefly, the full-length 16S rRNA gene was amplified using the PacBio uni-tagged primers 27F (5’-gcagtcgaacatgtagctgactcaggtcacAGRGTTYGATYMTGGCTCAG-3’) and 1492R (5’-tggatcacttgtgcaagcatcacatcgtagRGYTACCTTGTTACGACTT-3’). Asymmetric barcoding was performed using the uni-tagged PacBio forward barcodes 1F-4F and reverse barcodes 16R-30R. Barcoded amplicons were pooled in equimolar concentrations and gel purified using a QIAGEN Gel Extraction Kit. Samples were sequenced using PacBio single molecule HiFi sequencing on a single SMRT cell at the Ramaciotti Centre for Genomics (NSW, Australia).

### Raw sequence analysis

Raw data were processed at the Ramaciotti Centre for Genomics using PacBio SMRTLink analysis software v6.0 to generate demultiplexed.fastq files. Demultiplexed HiFi reads were filtered, aligned, clustered, and taxonomically assigned using Mothur v.1.44.1 [49] against the SILVA reference database (v138) [50] as previously described [48]. Rarefaction was performed based on the smallest library size (1007 reads).

### Statistical analyses

Alpha diversity was measured using richness (number of different OTUs) and Shannon diversity. Differences in alpha diversity measures between maternal and infant characteristics were assessed using Wilcoxon Rank Sum tests for categorical characteristics and Kruskal Wallis tests for continuous characteristics. A continuity correction was included in the Wilcoxon Rank Sum tests to account for ties in richness. Differences in beta diversity between the categorical and continuous maternal and infant characteristics were assessed through univariate PERMANOVAs on Bray-Curtis distances. These analyses were performed using the R environment for statistical computing [51]. To analyse whether any OTUs were differentially abundant based on maternal or infant factors, the metastats tool [52] was used in Mothur v.1.44.1 [49]. The relative abundance of OTUs was compared across the groups by conducting a two-sample t-test. OTUs of interest were mapped taxonomically using BLAST [53, 54] with a sequence identity score of > 97.8% being considered a good match. Significant results were reported only for OTUs with an average relative abundance of ≥ 1% across all samples that were present in > 1 sample. For all tests, p-values < 0.05 were considered significant. Bonferroni correction was applied to the significance level.

## Results

**Fig 1:**
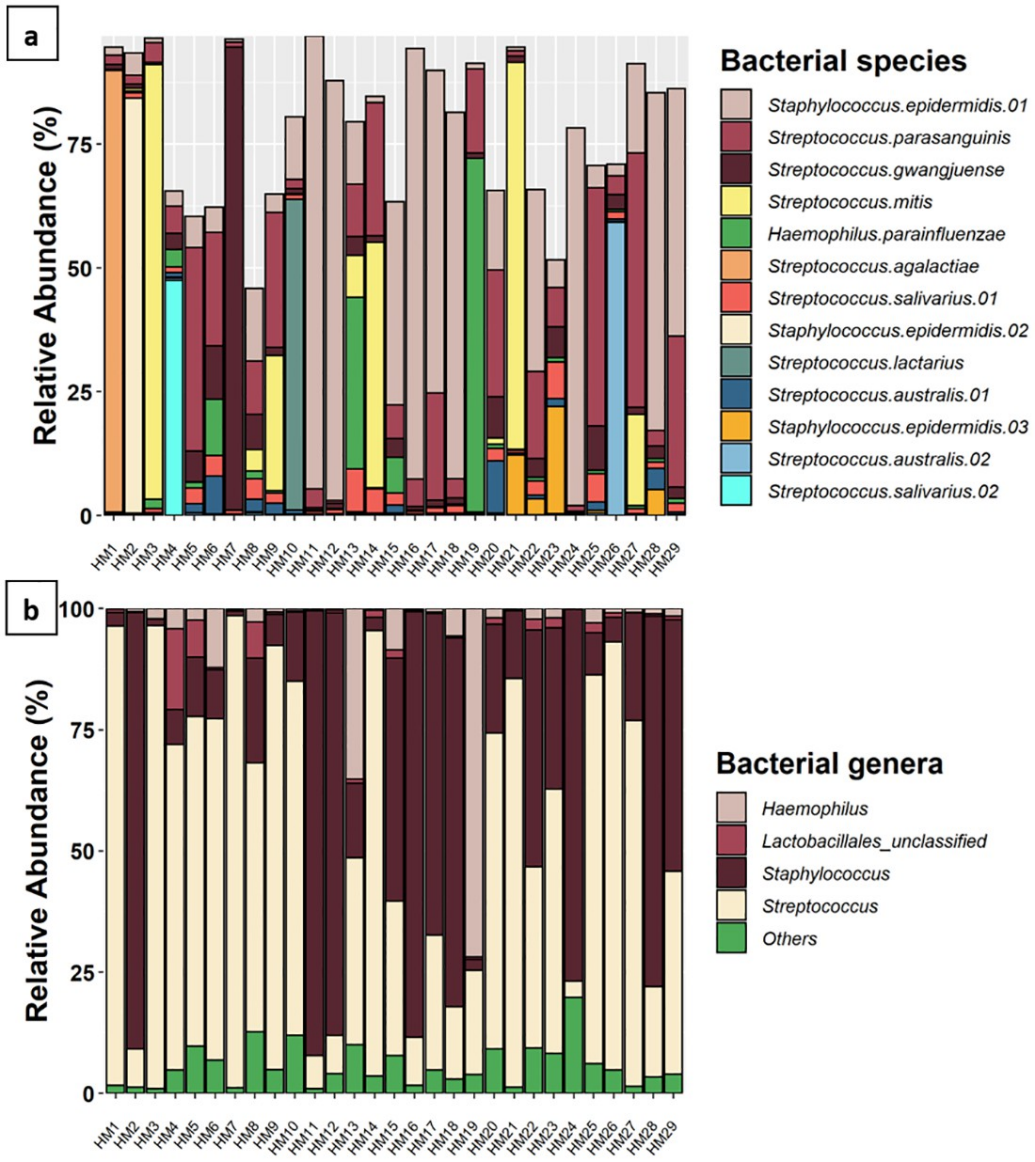
The relative abundance of bacterial OTUs and genera in HM samples. (a) The relative abundance of OTUs which made up ≥1% overall relative abundance. Species assigned to each OTU are noted in the legend. Where multiple OTUs mapped to the same species, they are numbered. (b) The relative abundance of bacterial genera which made up ≥ 1% relative abundance across all samples. Genera which accounted for < 1% relative abundance are grouped together as “others”.

Characteristics of the 29 mothers and their infants are included in Table 1. 13 OTUs made up ≥1% relative abundance in these HM samples. The five most abundant OTUs mapped to *Staphylococcus epidermidis* (mean relative abundance: 27.3%), *Streptococcus parasanguinis* (14.0%), *Streptococcus mitis* (9.5%), *Streptococcus gwangjuense* (6.2%), and *Haemophilus parainfluenzae* (4.9%) (Fig 1a). At the genus level, *Streptococcus* spp. (52.3%) and *Staphylococcus* spp. (34.7%) dominated the profiles (Fig 1b).

**Table 1:** Participant characteristics (*n* = 29).

Variables	Mean (range) or n (%)
Maternal age (years)	32.8 (24–40)
Maternal ethnicity:^a^	
Caucasian	26 (89.6%)
Aboriginal Australian	1 (3.4%)
Maternal BMI^b^	25.1 (16.9–38.3)
Obesity class:	
Normal	13 (44.8%)
Overweight	9 (31%)
Obesity class II	3 (10.3%)
Underweight	3 (10.3%)
Primiparous	15 (51.7%)
Maternal allergy	11 (37.9%)
Previous mastitis^c^	4 (13.7%)
Breast pump use	24 (82.7%)
Mode of delivery:^b^	
Vaginal	19 (65.5%)
Emergency caesarean section	5 (17.2%)
Elective caesarean section	4 (13.7%)
Current maternal antibiotic intake^a^	1 (3.4%)
Infant age (weeks)	23.3 (3.4–83.3)
Gestational age at delivery (weeks)	39.2 (35–41)
Male infant	16 (55.1%)
Mode of feeding:	
Exclusive breastfeeding	15 (51.7%)
Human milk and formula	4 (13.7)
Human milk and solids	10 (34.4%)
Pacifier use	15 (51.7%)

^a^ missing variable value for two participants

^b^ missing variable value for one participant

^c^ none of the mothers presented with symptoms of mastitis at the time of milk sample collection

### HM bacterial profiles are associated with maternal and infant factors

Differences in HM bacterial composition were observed relative to maternal BMI, mode of delivery, breast pump use, and pacifier use. However, lactation stage, maternal allergy, parity, infant sex, mode of feeding, and solid food intake showed no significant relationship with the HM bacterial profiles.

#### Maternal BMI

**Fig 2:**
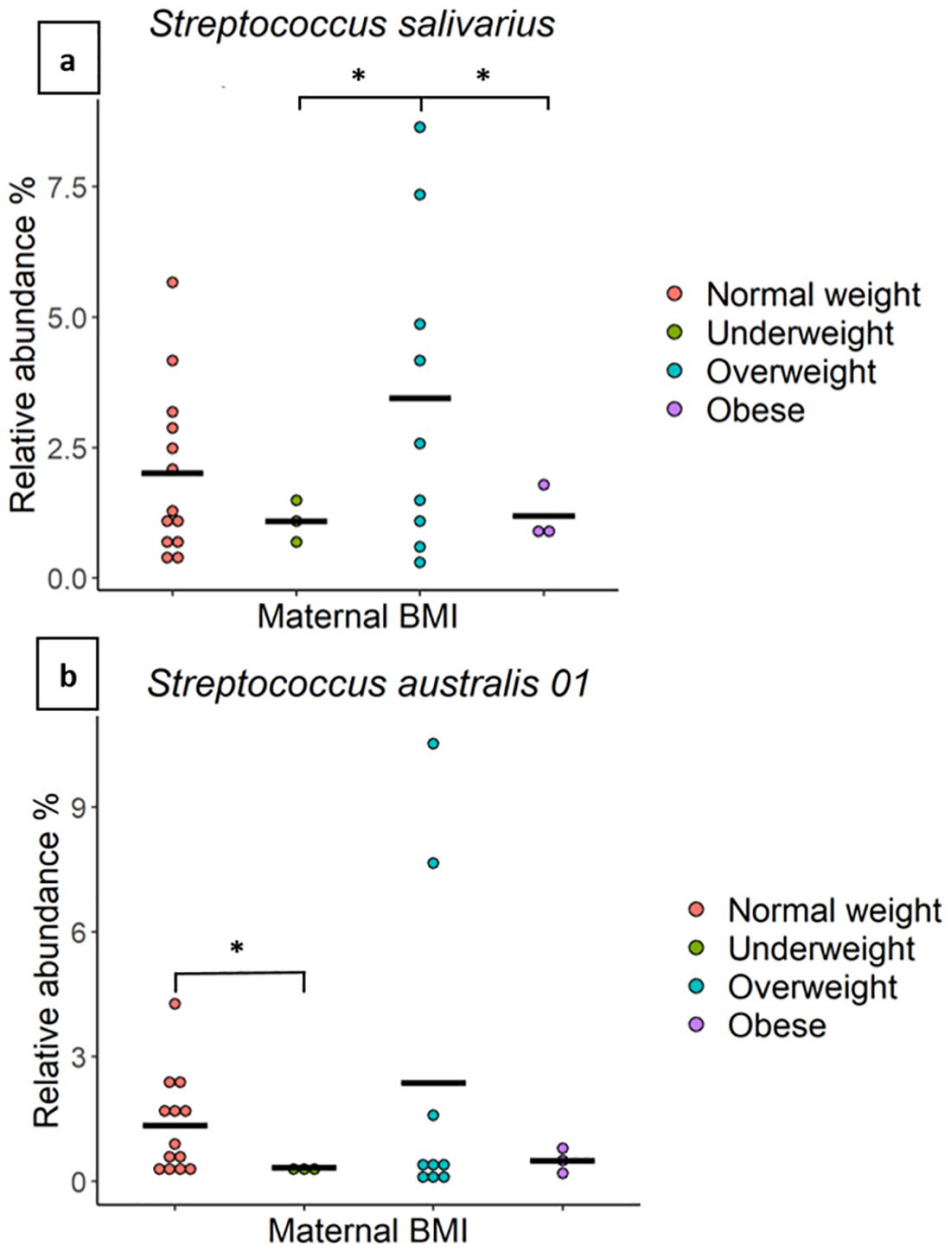
The relative abundance of *Streptococcus* spp. is associated with maternal BMI. The relative abundance of two OTUs, mapping to (a) *Streptococcus salivarius* and (b) *Streptococcus australis* in milk from mothers of different BMI classes (normal weight n = 13, overweight n = 9, obese n = 3, and underweight n = 3). The average value for each group is indicated with a line. * indicate significant results.

Milk of overweight mothers (n = 9) showed a significantly higher relative abundance of an OTU which mapped to *Streptococcus salivarius* compared to underweight mothers (n = 3) (mean relative abundance: 3.45% vs 1.09%, *P* = 0.03) and obese mothers (n = 3) (3.45% vs 1.19%, *P* = 0.04) (Fig 2a). Additionally, milk from normal weight mothers (n = 13) showed a significantly higher relative abundance of *Streptococcus australis* compared with milk from underweight mothers (1.34% vs 0.33%, *P* = 0.02) (Fig 2b). No differences in richness, beta diversity, or Shannon diversity were detected based on maternal BMI.

#### Mode of delivery

**Fig 3:**
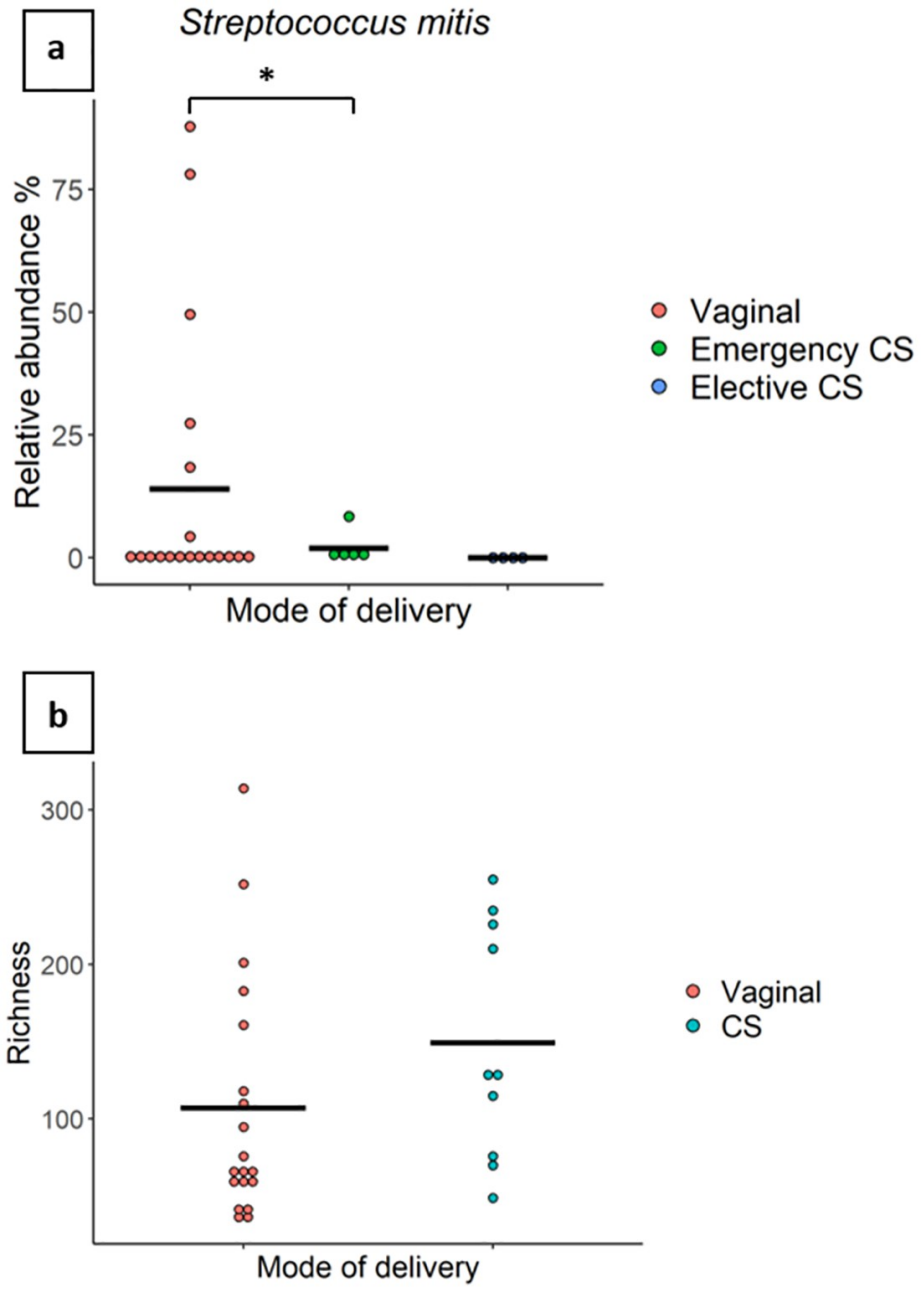
The relative abundance of *Streptococcus mitis* and bacterial richness are associated with the mode of delivery. (a) The relative abundance of *Streptococcus mitis* in milk from mothers who delivered vaginally (n = 19), via emergency caesarean section (n = 5), or via elective caesarean section (n = 4). The average value for each group is indicated with a line. (b) Richness of milk samples from mothers who delivered via caesarean section (n = 10) or vaginally (n = 19). * indicate significant results.

Milk from mothers who delivered vaginally (n = 19) had a significantly higher relative abundance of an OTU which mapped to *S*. *mitis* (14.01% vs 1.95%, *P* = 0.05) (Fig 3a) than that of mothers who delivered via emergency caesarean section (CS) (n = 5). Although no significant differences were detected in Shannon or beta diversities based on mode of delivery, HM from mothers who delivered via CS (n = 10) showed a trend toward a higher bacterial richness compared to those who delivered vaginally (n = 19), however, this did not reach statistical significance (*P* = 0.07) (Fig 3b).

#### Breast pump use

**Fig 4:**
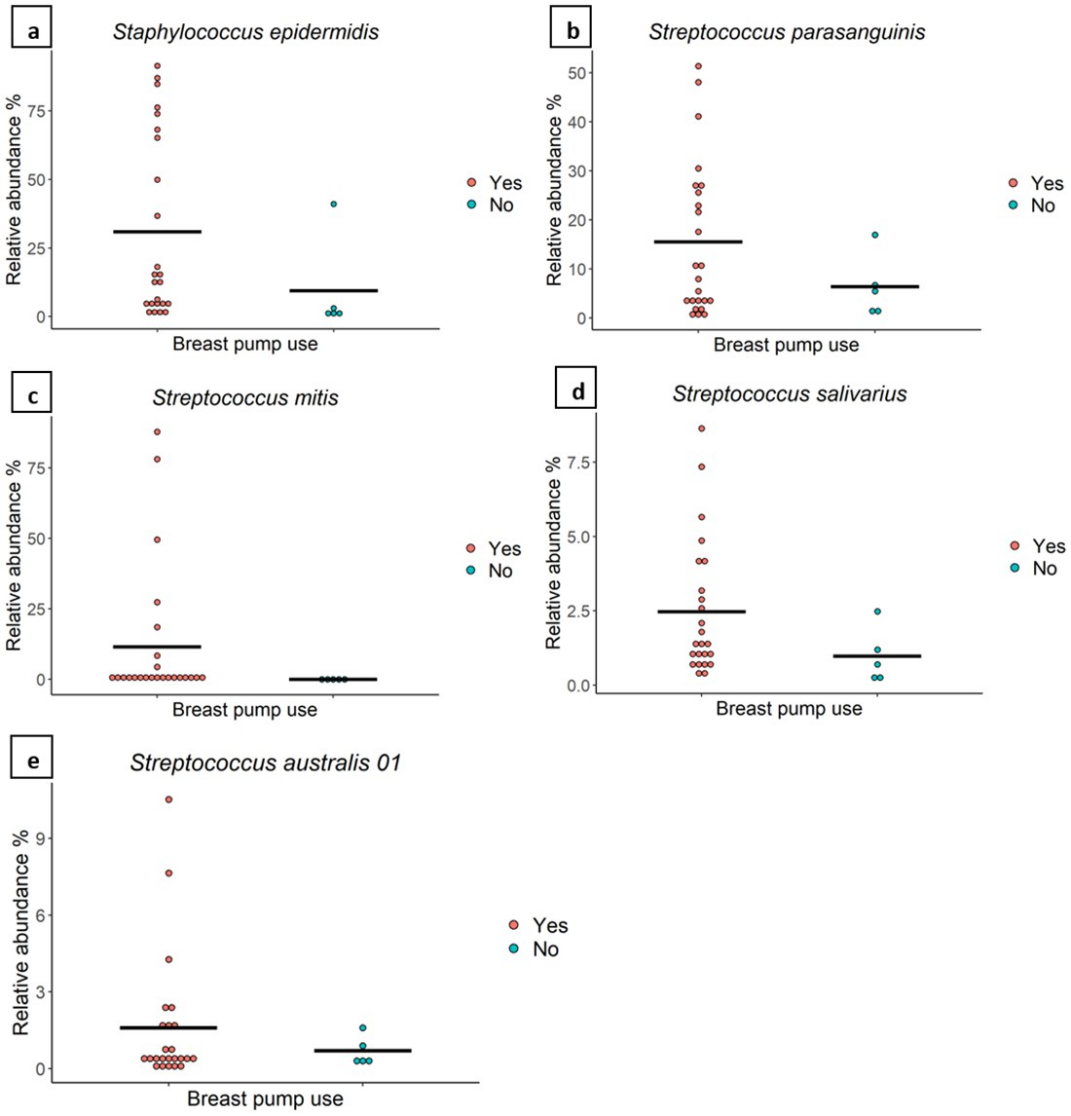
The relative abundance of *Streptococcus* spp. and *Staphylococcus* sp. is associated with breast pump use. The relative abundance of various bacterial taxa in milk from mothers who did (n = 24) or did not (n = 5) use a breast pump. The average value for each group is indicated with a line.

Milk of mothers who used a breast pump (n = 24) had a higher relative abundance of OTUs which mapped to *S*. *epidermidis* (30.95% vs 9.51%, *P* = 0.000002), *S*. *parasanguinis* (15.52% vs 6.42%, *P* = 0.000002), *S*. *mitis* (11.50% vs 0%, *P* = 0.000002), *S*. *salivarius* (2.47% vs 0.97%, *P* = 0.000002), and *S*. *australis* (1.59% vs 0.69%, *P* = 0.000002) compared to milk of mothers who did not use a breast pump (n = 5) (Fig 4). Richness, beta diversity, and Shannon diversity did not differ according to breast pump use.

#### Pacifier use

**Fig 5:**
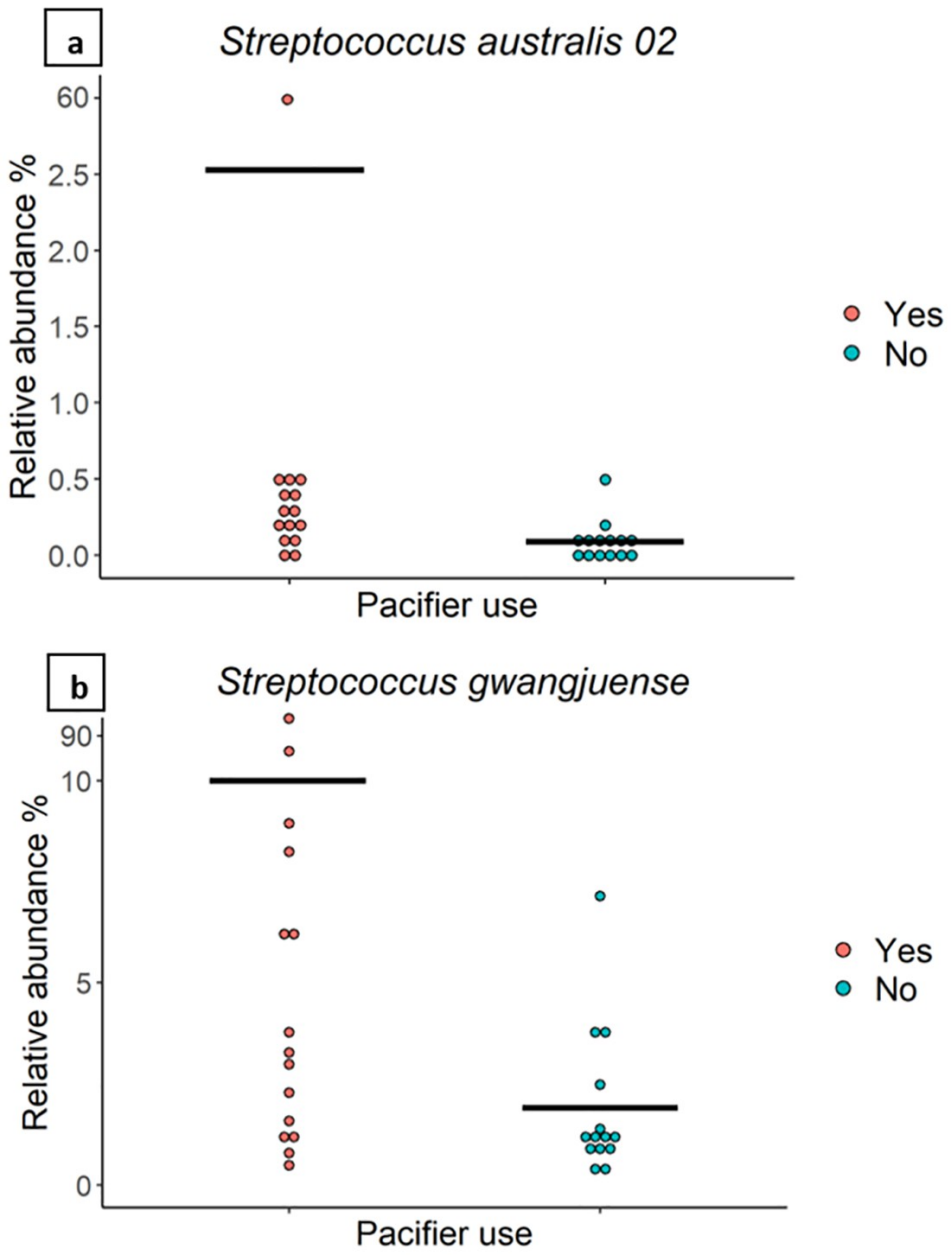
The relative abundance of *Streptococcus* spp. is associated with pacifier use. The relative abundance of bacterial taxa in milk from mothers whose infants did (n = 15) or did not (n = 14) use a pacifier. The average value for each group is indicated with a line.

Milk of mothers whose infants used a pacifier (n = 15) had a significantly higher relative abundance of OTUs which mapped to *S*. *australis* (4.19% vs 0.09%, *P* = 0.003) and *S*. *gwangjuense* (10.10% vs 1.92%, *P* = 0.02) compared to that of mothers whose infants had never used a pacifier (n = 14) (Fig 5). Richness, beta diversity, and Shannon diversity did not differ by pacifier use.

#### Lactation stage

No significant differences in the relative abundance of the most abundant bacterial species (relative abundance of ≥ 1%) were detected based on samples collected < 6 months postpartum (n = 19) and those collected > 6 months postpartum (n = 10). Richness (113.5 ± 82.1, 137.8 ± 75.5, *P* = 0.2), beta diversity (PERMANOVA, *P* = 0.5) (S1 Fig), and Shannon diversity (1.7 ± 1.1, 2.3 ± 0.9, *P* = 0.1) did not differ significantly by lactation stage.

## Discussion

In this pilot study, we identified maternal BMI, mode of delivery, breast pump use, and pacifier use as factors that were associated with HM bacterial composition. While similar studies have been performed elsewhere in the world [27–31, 33, 34, 36–39, 55], this is the first study to assess such associations in an Australian population. This is important given that the HM microbiome has been shown to vary geographically (due to a combination of genetic, local environment, and dietary factors) [3, 26, 30]. Further, by using full-length 16S rRNA gene sequencing, we have been able to resolve these associations to the species level, whereas previous such studies have been limited to the genus or family level [27, 34, 42].

Several studies have reported an association between maternal BMI and the HM microbiome [27, 30, 35–38]. In the present cohort, milk from overweight mothers was associated with a higher abundance of *S*. *salivarius* compared to milk from obese mothers (3.45% vs 1.19%) and underweight mothers (3.45% vs 1.09%). Additionally, we showed that milk from normal weight mothers had a higher abundance of *S*. *australis* compared with milk from underweight mothers (1.34% vs 0.33%) (Fig 2). These findings may be reflective of BMI-related differences in the human microbiome [56–61] or differences in maternal diet [38, 62–65]. However, with only three overweight and three underweight mothers in this study, the results should be interpreted with caution. High maternal BMI has been associated with a higher abundance of *Staphylococcus* spp. [35], *Akkermansia* spp. [27, 36], and *Granulicatella* spp. [38], and a decreased abundance of *Bifidobacterium* spp. [27, 36], *Lactobacillus* spp., and *Streptococcus* spp. [35] in HM. In addition, milk from mothers with a high BMI has been reported to have a lower bacterial diversity and a higher total bacterial count [27]. We could not identify such changes in the present study, potentially due to population and methodologic differences. Nevertheless, these preliminary results suggest that maternal BMI is associated with HM bacterial composition in an Australian cohort.

Mode of delivery has been repeatedly associated with the composition of the HM microbiome [27–35]. We found a higher abundance of the typical oral taxa *S*. *mitis* [66] (14.01% vs 1.95%) in milk from mothers who delivered vaginally compared to those who delivered via emergency CS (Fig 3a). The frequency of breastfeeding may play a role in the increase of oral bacteria in HM of women who delivered vaginally, as CS delivery is associated with more breastfeeding difficulties [67]. Unfortunately, we do not have a complete data set on breastfeeding difficulties in this cohort. Moreover, in Australia, intrapartum antibiotic prophylaxis is administered during CS. Antibiotics can induce changes to the maternal gut microbiome [68, 69], which may affect HM bacteria via the enteromammary route [4, 5, 10, 11]. Antibiotics can also have a direct effect on the HM microbiome [33]. Hermansson et al. provided evidence that exposure to intrapartum antibiotics is associated with alterations to the HM microbiome, regardless of delivery mode [33]. Therefore, the observed associations between HM bacterial composition and CS delivery may be, at least in part, driven by intrapartum antibiotic administration.

Milk from mothers who habitually used a breast pump (on a daily or almost daily basis) was associated with a significantly higher relative abundance of typical oral taxa including *S*. *parasanguinis* (15.52% vs 6.42%) [70], *S*. *salivarius* (2.47% vs 0.97%) [71], *S*. *mitis* (11.50% vs 0%) [66], and *S*. *australis* (1.59% vs 0.69%), as well as the typical skin taxon *S*. *epidermidis* (30.95% vs 9.51%) compared to the milk of mothers who did not use a breast pump (Fig 4). It is unclear why these taxa were at a higher relative abundance in milk from mothers who used a breast pump; however, the high abundance of *S*. *epidermidis*, may be related to the disturbance of skin bacteria during the application of vacuum during pumping, allowing easier entry through the nipple to the mammary gland. Analysis of samples collected from nipples and areola before sterilisation might provide some evidenc. More research is needed to confirm whether bacteria enter the mammary gland through the nipple or whether milk inoculation occurs as milk is expressed. In addition, cleaning practices of breast pump parts between each pumping session could have contributed to the observed differences. Steam decontamination of the breast pump kit has been reported to significantly decrease milk contamination with Enterobacteriaceae and *Candida* spp. compared with milk samples collected with breast pump accessories that were only washed and not decontaminated [72]. Further research should be performed to investigate whether the observed differences in milk associated with use of a breast pump originate from pump cleaning practices. We are not the first to report that breast pump use is associated with alterations to the HM microbiome. Fehr et al. reported that HM of mothers who sometimes used a breast pump had a significantly lower relative abundance of *Veillonella dispar*, *Haemophilus parainfluenzae*, *Streptococcus* spp., and *Bifidobacterium* spp. compared with those who only directly breastfed their infants [39]. In contrast to our results, Moosavi et al. reported that direct breastfeeding was associated with a higher abundance of Actinobacteria and *Veillonellaceae*, while the use of a breast pump at least once during the last two weeks was associated with the presence of potential opportunistic pathogens such as *Stenotrophomonas* spp. and *Pseudomonadaceae* [34].

Infant pacifier use was associated with a significantly higher relative abundance of *S*. *australis* (4.19% vs 0.09%), a species first isolated from the oral cavities of Australian children in 2001 [66], and *S*. *gwangjuense* (10.10% vs 1.92%), a species first isolated from human pericoronitis, an inflammation of the periodontal tissue surrounding unerupted teeth [73] (Fig 5). To our knowledge, this is the first cohort in which *S*. *gwangjuense* has been identified in HM. Sharing of bacterial taxa between the infant oral cavity and HM has been repeatedly demonstrated in previous studies [9, 10, 41]. Retrograde flow occurs during breastfeeding, with HM from the infant’s mouth flowing back through the milk ducts to the alveoli during the second half of milk ejection as oxytocin is degraded and intraductal pressure reduces [74].

Pacifier use has been shown to be associated with differences in infant oral microbiome composition. Two studies have reported a significant positive association between pacifier use and the count of microbes such as lactobacilli and *Candida* spp. in the infant oral microbiome [75, 76]. Other studies reported a significantly higher prevalence of *Candida* spp. colonisation in the oral microbiota of infants who use a pacifier compared with those who did not [77, 78]. Pacifier use may influence oral sugar clearance in a manner similar to removable dentures, which have been implicated in less effective clearance [79]. This would prolong low pH conditions, making the oral cavity favourable to aciduric microorganisms [80, 81]. In this manner, pacifier use may alter the composition of the oral microbiota and thereby influence microbes contributing to the HM microbiome through retrograde flow. Thus, this study provides preliminary evidence that pacifier use is associated with the HM bacterial profile; however, larger studies are required to replicate these findings with the addition of information on frequency of pacifier use and pacifier cleaning practices.

We did not identify significant associations between maternal allergy, parity, infant sex, mode of feeding, and introduction of solids and the bacterial composition of HM. For parity and infant sex, this is supported by previous findings from Williams et al. [38] and Urbaniak et al. [24], respectively. In contrast, one large cohort study reported an association between maternal atopy, parity, and infant sex and HM bacterial composition [34]. Multiparous mothers and those with atopy had a higher Actinobacteria richness in their milk, while mothers with male infants had a decreased bacterial richness [34]. However, milk samples were not collected aseptically, which may have contributed to the observed differences in results. In a different study, mothers with male infants had an increased relative abundance of *Streptococcus* spp. and a lower relative abundance of *Staphylococcus* spp. in their milk [38]. Only one study has investigated the association between exclusive breastfeeding and the HM microbiome, and this was in an area of high HIV prevalence. Milk from mothers who fed their infants exclusively showed an increased relative abundance of *Streptococcus parasanguis* than those who used mixed feeding methods [40]. These contradictory results could be attributed to the use of short amplicon sequencing, geographically different populations (genetic, local environment, and dietary factors), sample size, and use of non-sterile sample collection techniques by these studies.

A number of limitations in this pilot study need to be acknowledged. The major limitation of this study is the small sample size, which limits the generalisability of these results and their relevance to infant health. Further study in larger cohorts may strengthen the conclusions drawn from this pilot study. Future studies with larger sample sizes will also be able to employ multivariable analyses. Milk samples were collected from participants over a large range of lactation stages (mean: 23.4 weeks ± 18.9 weeks, range 3.4–83.3 weeks, median: 17.3 weeks) among the 29 milk samples. The study is also limited by the lack of information on maternal breast pump cleaning practices, intrapartum antibiotic administration, frequency of breastfeeding, and breastfeeding difficulties.

## Conclusions

The current study demonstrates that maternal BMI, mode of delivery, breast pump use, and pacifier use are significantly associated with the bacterial composition of HM in a small cohort of exclusively breastfeeding Western Australian women. The association of these factors with HM bacterial profiles highlights the importance of both mother and infant as contributors to the HM microbiome; however, these conclusions remain statistically insignificant. Therefore, further research is needed to investigate and validate these relationships in a larger cohort and determine if these relationships are related to infant health and development.

## Supporting information

S1 Fig PCoA of Bray-Curtis distances between human milk samples collected < 6 months postpartum or > 6 months postpartum. (TIF)
