# Supplementary figures and images for: Characterisation of human milk bacterial DNA profiles in a small cohort of Australian women in relation to infant and maternal factors
Source: PLoS One. 2023 Jan 25;18(1):e0280960. doi: 10.1371/journal.pone.0280960 (PMC9876237; doi:10.1371/journal.pone.0280960)

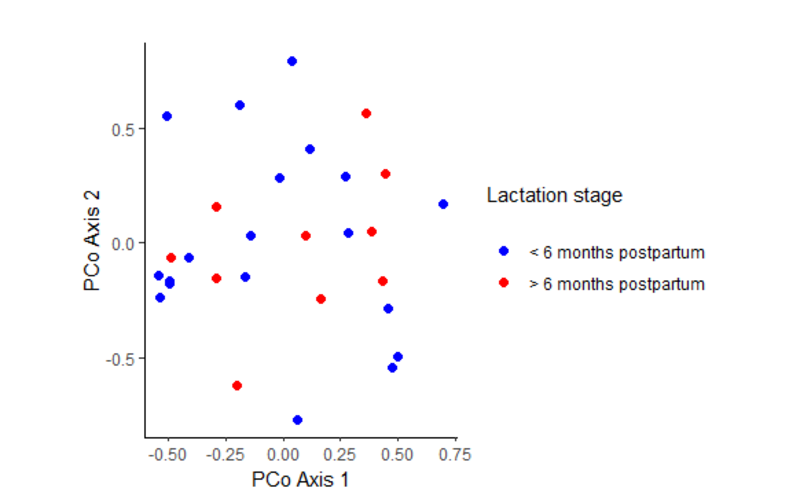

Supplement: S1 Fig — (TIF) [file pone.0280960.s001.tif]
